# Thymoma complicated with myasthenia gravis and Good syndrome – a therapeutic conundrum: a case report

**DOI:** 10.1186/s13256-019-2289-z

**Published:** 2019-11-29

**Authors:** Shiran Paranavitane, Sumana Handagala, Rajiva De Silva, Thashi Chang

**Affiliations:** 1 University Medical Unit, National Hospital of Sri Lanka, Colombo, Sri Lanka; 2 National Hospital for Respiratory Diseases, Welisara, Sri Lanka; 3 Department of Immunology, Medical Research Institute, Borella, Sri Lanka; 4 Department of Clinical Medicine, Faculty of Medicine, University of Colombo, Colombo, Sri Lanka

**Keywords:** Thymoma, Myasthenia gravis, Good syndrome, Sri Lanka

## Abstract

**Background:**

Thymomas are known to be associated with myasthenia gravis and Good syndrome. Good syndrome is the association of thymoma with combined B cell and T cell immunodeficiency. The combination of all three diseases has not been reported. We discuss the therapeutic dilemma of immunosuppression in such a case.

**Case presentation:**

A 27-year-old Sinhalese man was evaluated for persistent cough which was associated with pleuritic chest pain and was found to have pleural-based lesions in his left hemithorax. Further evaluation confirmed these lesions to be implants from a thymoma. He subsequently developed myasthenia gravis and impending myasthenic crisis precipitated by pneumonia. He was found to have hypogammaglobulinemia with low B cell counts, confirming a diagnosis of Good syndrome. Treatment with intravenously administered broad-spectrum antibiotics, acetylcholinesterase inhibitors, orally administered glucocorticoids, plasma exchange, and intravenous immunoglobulin led to clinical improvement. He subsequently underwent thymectomy and debulking of the tumor and was maintained on regular intravenous immunoglobulins combined with low-dose prednisolone.

**Conclusions:**

Regular intravenous immunoglobulins combined with low-dose immunosuppression in addition to thymectomy appear to be safe when myasthenia gravis occurs in association with Good syndrome.

## Background

Thymoma is the most common neoplasm arising from the thymus [1]. Thymomas have been reported to be associated with several parathymic syndromes such as myasthenia gravis (MG) and pure red cell aplasia [2]. Approximately, 30–50% of patients with thymomas develop MG [3].

Good syndrome is the association of thymoma with immunodeficiency [4]. It is a rare cause of combined B cell and T cell immunodeficiency, which was first recognized in 1954 [4]. Around 0.2–6% of thymomas are associated with Good syndrome [3]. MG is characterized by fatigable muscle weakness causing potentially fatal respiratory paralysis. The mainstay of treatment in MG is immunosuppression and immunomodulation.

We report a case of thymoma associated with MG and Good syndrome and discuss the therapeutic dilemma of whether it is safe to immunosuppress and, if it is safe, how best to do that for the treatment of MG when it occurs in association with Good syndrome, and review the relevant literature.

## Case presentation

A 27-year-old Sinhalese man presented with a 2-year history of intermittent left-sided pleuritic-type chest pain which was associated with a non-productive cough and wheezing. He was previously healthy and self-employed. He did not have a family history of note and denied smoking tobacco or consuming alcohol.

**Fig. 1:**
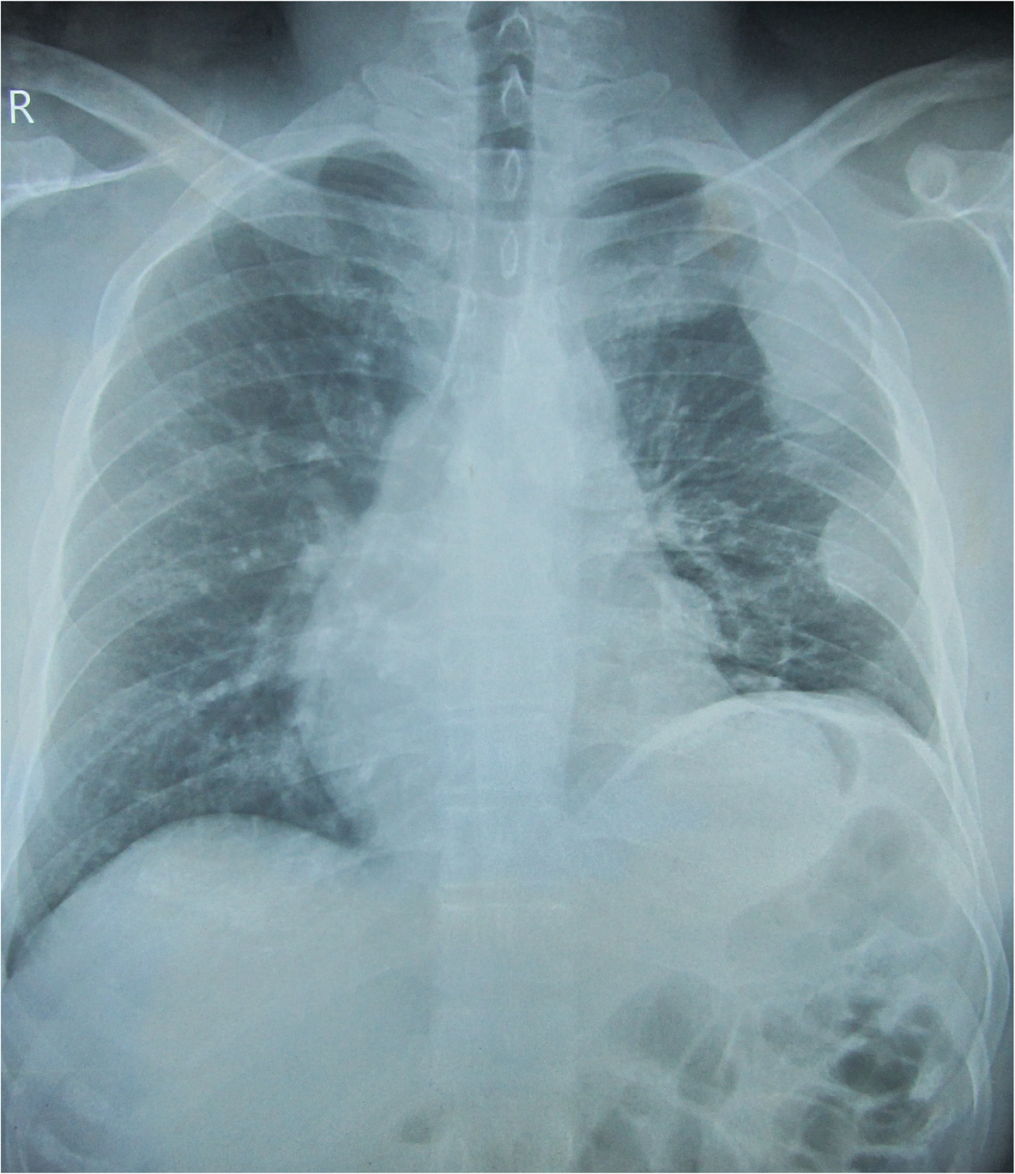
Chest X-ray posteroanterior view showing pleural-based deposits on the left side

**Fig. 2:**
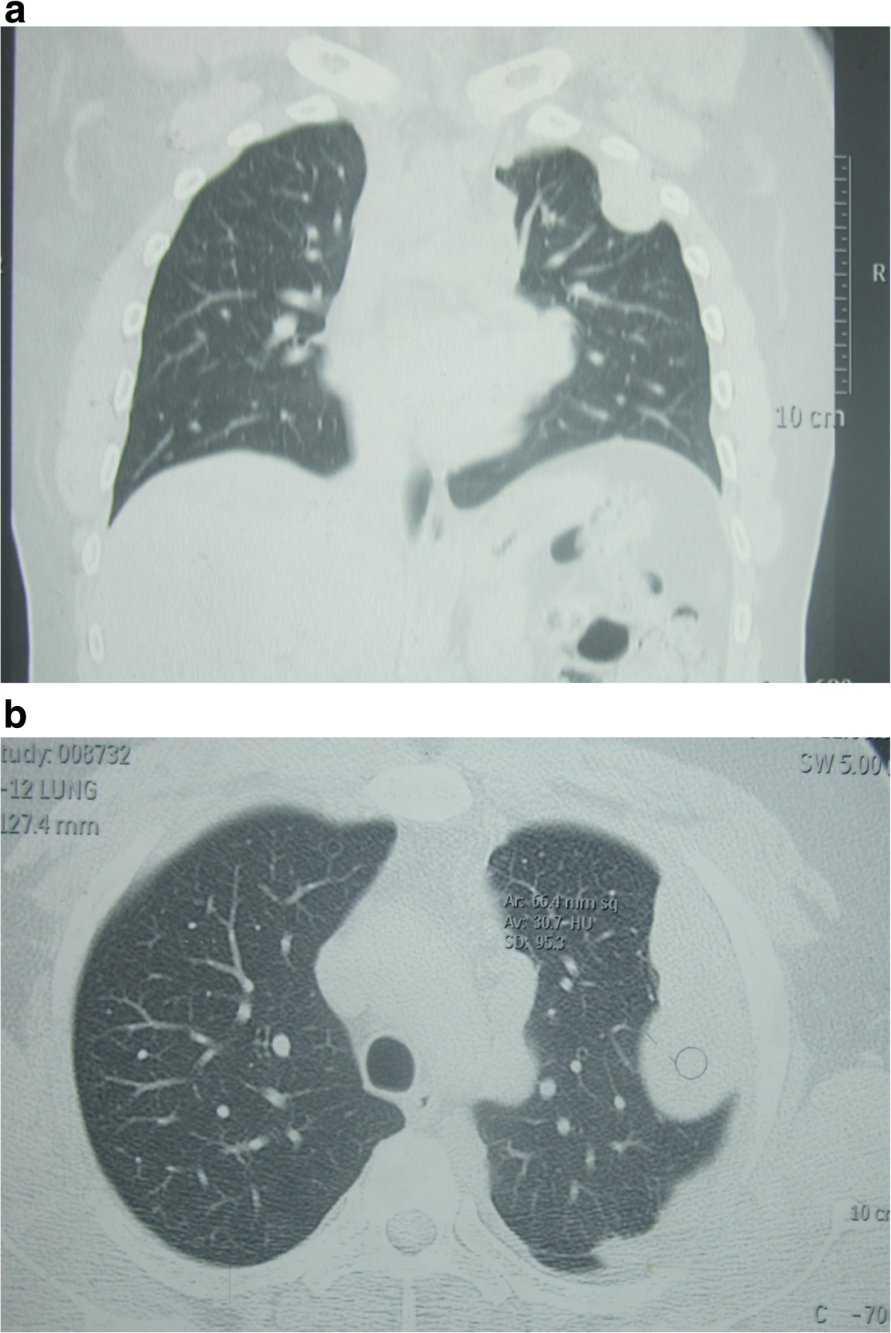
Contrast-enhanced computed tomography of the chest showing mediastinal enlargement and pleural-based deposits. **a** Coronal view. **b** Axial view

He had been treated with inhaled bronchodilators, inhaled corticosteroids, and antibiotics intermittently. During evaluation, his chest X-ray revealed a pleural-based lesion along the lateral wall of his chest with lobulated inner margins in the left hemithorax (Fig. 1). Further evaluation with a contrast-enhanced computed tomography (CT) scan of his chest revealed multiple pleural-based enhancing focal lesions involving the left hemithorax with calcifications (Fig. 2). A CT-guided Tru-Cut biopsy confirmed the diagnosis of a thymoma. While awaiting thymectomy and debulking surgery, he developed diplopia with a right-sided abducens nerve palsy and partial ptosis on the same side. An MRI of his brain with orbits was normal. Repetitive nerve stimulation of facial and spinal accessory nerve-muscle pairs showed significant decrement and his acetylcholine receptor (AChR) antibody titer was 11.8 nmol/L (normal < 0.4 nmol/L), thus, confirming the clinical diagnosis of MG.

Prior to starting immunosuppressive treatment for MG, he developed fever with chills and rigors which was associated with progressively worsening difficulty in breathing and a productive cough over a period of 1 week.

An initial examination found bilateral, asymmetrical partial ptosis and a right sixth cranial nerve palsy. Demonstrable muscle fatigability was present. His neck flexion power on admission was grade 4/5. Upper and lower limb examinations were neurologically normal. However, his forced vital capacity (FVC) on admission was 1.0 liter. In addition, he had clinical features consistent with a left lower zone pleural effusion without tracheal deviation. The rest of his physical examination was normal.

Pneumonia of the lower lobe of his left lung with parapneumonic effusion and impending myasthenic crisis were diagnosed and he was commenced on intravenously administered meropenem 1 g every 8 hours, after taking blood and sputum for microbiological cultures. He was treated with orally administered pyridostigmine 60 mg 6 hourly for the fatigable weakness.

A summary of his laboratory parameters are shown in Table 1.

**Table 1:** Summary of the hematological and biochemical parameters

Laboratory parameter	Value	Reference range
Hematology		
Total white cell count (cells/microL)	15,490	4000–11,000
Neutrophil count (cells/microL)	12,740	1500–8000
Lymphocyte count (cells/microL)	1800	1000–4800
Hemoglobin level (g/dL)	14.9	13.5–17.5
Platelet count (platelets/microL)	364,000	150,000–450,000
Erythrocyte sedimentation rate (mm/first hour)	101	
Biochemistry		
C-reactive protein (mg/L)	258	< 6
Sodium (mmol/L)	135	135–148
Potassium (mmol/L)	4.2	3.5–5.1

His chest X-ray showed left lower zone consolidation with a pleural effusion which was confirmed on an ultrasound scan of his chest.

On day 3 of hospital stay plasmapheresis was commenced. On day 4 of hospital stay, there was worsening bulbar weakness and neck flexion and he required admission to the intensive care unit (ICU). In addition, orally administered prednisolone was commenced at 10 mg daily and increased by 10 mg per day until a dose of 60 mg daily was reached, which was then continued. His serum potassium levels, C-reactive protein, and white cell counts were monitored.

A total of five cycles of plasma exchanges were performed on an every-other-day basis and subsequently intravenous immunoglobulin at a dose of 0.4 g/kg per day for 5 days was given. Intravenously administered meropenem was continued for 14 days. He did not require endotracheal intubation or mechanical ventilation.

During the ICU stay he was detected to have low immunoglobulin G (IgG) and immunoglobulin M (IgM) levels. Flow cytometry analysis of lymphocyte subsets revealed low B cell counts (Table 2).

**Table 2:** Summary of the immunological parameters (peripheral blood)

Laboratory parameter	Value	Reference range
Total protein (g/L)	55	61–80
Albumin (g/L)	35.5	36–50
Globulin (g/L)	19.5	22–40
IgG levels (mg/dL)	466	569–1919
IgA levels (mg/dL)	144	61–330
IgM levels (mg/dL)	43	47–147
CD19 (cells/microL)	97	100–500
CD8 (cells/microL)	154	200–900
CD4 (cells/microL)	417	387–1256

*CD* cluster of differentiation, *IgA* immunoglobulin A, *IgG* immunoglobulin G, *IgM* immunoglobulin M

Good syndrome was diagnosed due to the presence of hypogammaglobulinemia and thymoma. Following completion of intravenous immunoglobulin therapy, he underwent thymectomy and debulking of thymic tumor deposits. Postoperatively, he made a good recovery without any episodes of acute weakness or the development of infections. Histology of the surgical specimens revealed type B2 thymoma with implants in his left lung and parietal pleura. His prednisolone dosage was gradually tapered to a maintenance dose of 10 mg/day without relapse of MG at 6-month follow-up.

## Discussion

We report a case of thymoma associated with MG and Good syndrome and discuss the therapeutic dilemma of using immunosuppressives in an already immunodeficient patient. To the best of our knowledge this combination of diseases and its inherent therapeutic dilemma has not been previously reported.

MG is an autoantibody-mediated disease involving the nicotinic receptors at the neuromuscular junction [5]. AChR antibodies, which are of the IgG1 and IgG3 subtypes, are the main antibodies found in patients with seropositive myasthenia, while a smaller proportion would have antibodies directed against tyrosine kinase muscle-specific kinase (MuSK) and low-density lipoprotein receptor-related protein 4 (LRP-4) [5]. In Sri Lanka, most patients with MG were found to be seropositive [6, 7]. Patients with MG are reported to have an associated thymoma in around 10% of patients [7, 8].

There are no randomized controlled studies performed regarding the management of Good syndrome. One review suggested that thymectomy and debulking of the tumor along with immunoglobulin replacement would be the best management option [4]. A review of five cases of Good syndrome showed that intravenous immunoglobulin replacement reduced the incidence of sinopulmonary infections [9].

MG is treated with drugs that bring about symptomatic improvement, such as acetylcholinesterase inhibitors and drugs that suppress the immune system. Among the immunosuppressive drugs, glucocorticoids are considered first-line agents [10]. In addition, azathioprine and mycophenolate mofetil are also used as first-line immunosuppressants [10]. Methotrexate, cyclosporine, and tacrolimus are considered alternate immunosuppressants [10].

Several agents have been used in treatment-refractory MG [11]. Thymectomy, rituximab, high-dose cyclophosphamide, and eculizumab are treatment modalities used in this situation [11]. Rituximab is a monoclonal antibody against CD20 molecule on B lymphocytes which leads to B lymphocyte depletion [11]. The efficacy of rituximab in a situation where the B lymphocytes are depleted as in Good syndrome is contentious. High-dose cyclophosphamide is known to substantially increase the risk of infections and long-term risk of malignancies [12]. In an immunodeficiency state such as Good syndrome, the use of cyclophosphamide may lead to an unacceptably high rate of infections.

Eculizumab is a monoclonal antibody that binds to C5 in the complement pathway and thereby preventing the activation of the final complement pathway involving the membrane attack complex [11]. This drug appears to be the least hazardous immunotherapy to a patient such as ours. However, in a resource-poor setting, the availability and exorbitant cost of eculizumab precludes its use.

Intravenous immunoglobulin together with plasma exchange has been used as a treatment modality in acute exacerbations of MG [13]. It has also been used as a form of intermittent maintenance therapy in the management of MG [14]. Our patient was placed on regular, 3 weekly intravenous immunoglobulin top ups in addition to low-dose orally administered prednisolone and pyridostigmine.

## Conclusions

From our experience with this patient, we think that in a patient who has undergone thymectomy for refractory MG and Good syndrome, regular intravenous immunoglobulin replacement, in addition to minimum orally administered immunosuppressants combined with anticholinesterases is an appropriate option. Furthermore, in the setting of B lymphocyte depletion, agents such as rituximab may not be effective and agents such as high-dose cyclophosphamide may pose a heightened risk of serious infections and are best avoided.

## Data Availability

All necessary data and material are provided.
